# Advances in Drug Resistance of Esophageal Cancer: From the Perspective of Tumor Microenvironment

**DOI:** 10.3389/fcell.2021.664816

**Published:** 2021-03-19

**Authors:** Siyuan Luan, Xiaoxi Zeng, Chao Zhang, Jiajun Qiu, Yushang Yang, Chengyi Mao, Xin Xiao, Jianfeng Zhou, Yonggang Zhang, Yong Yuan

**Affiliations:** ^1^Department of Thoracic Surgery, National Clinical Research Center for Geriatrics, West China Hospital, Sichuan University, Chengdu, China; ^2^West China Biomedical Big Data Center, West China Hospital, Sichuan University, Chengdu, China; ^3^Department of Periodical Press, National Clinical Research Center for Geriatrics, West China Hospital, Sichuan University, Chengdu, China; ^4^Nursing Key Laboratory of Sichuan Province, Chengdu, China

**Keywords:** esophageal cancer, drug resistance, tumor microenvironment, chemotherapy, targeted therapy, immunotherapy, chemoresistance, therapeutic response

## Abstract

Drug resistance represents the major obstacle to get the maximum therapeutic benefit for patients with esophageal cancer since numerous patients are inherently or adaptively resistant to therapeutic agents. Notably, increasing evidence has demonstrated that drug resistance is closely related to the crosstalk between tumor cells and the tumor microenvironment (TME). TME is a dynamic and ever-changing complex biological network whose diverse cellular and non-cellular components influence hallmarks and fates of tumor cells from the outside, and this is responsible for the development of resistance to conventional therapeutic agents to some extent. Indeed, the formation of drug resistance in esophageal cancer should be considered as a multifactorial process involving not only cancer cells themselves but cancer stem cells, tumor-associated stromal cells, hypoxia, soluble factors, extracellular vesicles, *etc*. Accordingly, combination therapy targeting tumor cells and tumor-favorable microenvironment represents a promising strategy to address drug resistance and get better therapeutic responses for patients with esophageal cancer. In this review, we mainly focus our discussion on molecular mechanisms that underlie the role of TME in drug resistance in esophageal cancer. We also discuss the opportunities and challenges for therapeutically targeting tumor-favorable microenvironment, such as membrane proteins, pivotal signaling pathways, and cytokines, to attenuate drug resistance in esophageal cancer.

## Introduction

Esophageal cancer (EC) is the sixth leading cause of cancer-related death globally, with a dismal overall 5-year survival of 20% (Lagergren et al., 2017; Bray et al., 2018). EC can be histologically classified as esophageal squamous cell carcinoma (ESCC) or esophageal adenocarcinoma (EAC), which has distinct pathogenesis, molecular characteristics, and geographical distribution (Cancer Genome et al., 2017; Lagergren et al., 2017). Clinically, chemotherapy has been one of the major therapeutic approaches in the trimodality therapy of EC; molecular targeted therapy and immune checkpoint inhibitors have been evaluating in preclinical and clinical trials (van Hagen et al., 2012; Smyth et al., 2017, 2021; Yang et al., 2020). However, despite recent advances in multidisciplinary management, the treatment of EC is still a relentless challenge partly owing to the fact that numerous patients are intrinsically insensitive or adaptively resistant to therapeutic agents. Indeed, over 70% of patients with locally advanced EC did not reach a pathological complete response (pCR) after the neoadjuvant chemoradiotherapy, ultimately leading to a risk of tumor relapse and poor prognosis (van Hagen et al., 2012). These patients suffered from unacceptable adverse-events and were delayed to surgery with virtually no therapeutic benefits. Definitive chemoradiotherapy, as an alternative to neoadjuvant chemoradiotherapy plus surgery, may offer a chance of cure. Unfortunately, a subset of these patients still require reintervention because of local relapse or distant metastasis (Swisher et al., 2002; Ilson and Lordick, 2018). To overcome these major challenges, the development of chemosensitizers and combined therapy is urgently needed, which requires further elucidation of the mechanism of drug resistance in EC.

The mechanism of cancer drug resistance is a complex multifactor process, including blockage of drug distribution, increased drug efflux, mutations of the drug target, DNA damage repair, activation of alternative pro-tumorigenic signaling pathways, evasion of programmed cell death, etc. (Holohan et al., 2013). This not only depends on malignant hallmarks of tumor cells, but also closely related to the aberrant state of the tumor microenvironment (TME) and the crosstalk between tumor cells and TME (Dalton, 1999). The solid tumor consists not only of cancer cells and cancer stem cells but also tumor-associated stromal cells (tumor-associated fibroblasts, immune and inflammatory cells, endothelial cells, etc.) and non-cellular elements (hypoxia, acidity, cytokines, extracellular matrix, exosomes, etc.), collectively defined as the TME (Hanahan and Weinberg, 2011; Hanahan and Coussens, 2012). Over the past few decades, increasing evidence has demonstrated that TME plays an important role in the initiation, progression, and therapeutic response of human cancer (Dalton, 1999; Trédan et al., 2007; Quail and Joyce, 2013). In this review, we mainly focus our discussion on molecular mechanisms that underlie the role of TME in drug resistance in EC. We also discuss the opportunities and challenges for therapeutically targeting tumor-favorable microenvironment, such as membrane proteins, pivotal signaling pathways, and cytokines, to attenuate drug resistance in EC.

## Cellular Components

### Cancer Stem Cells

**FIGURE 1:**
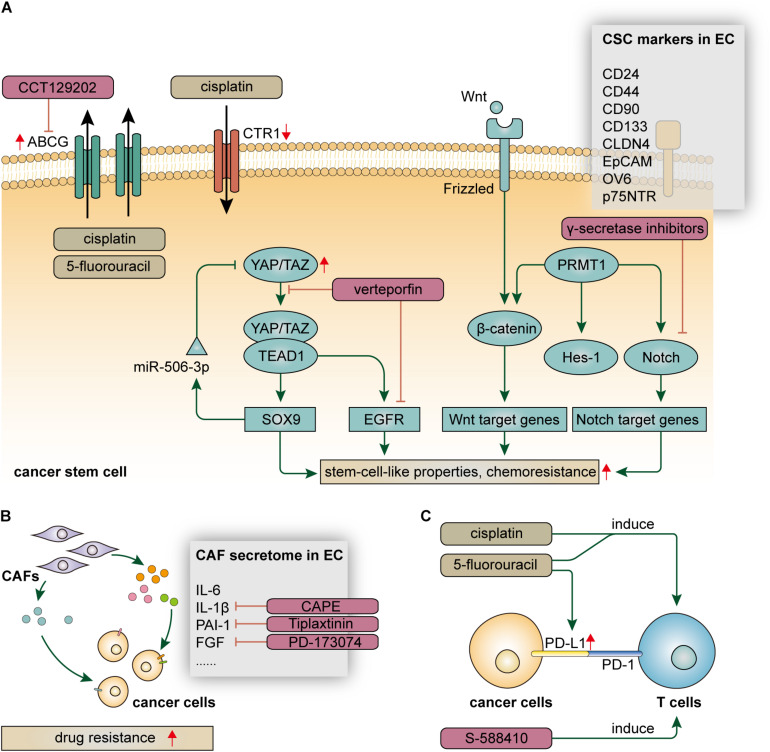
Resistance mechanisms of cellular components, including **(A)** cancer stem cells, **(B)** cancer-associated fibroblasts, and **(C)** immune inflammatory cells, in the tumor microenvironment in EC.

It is increasingly evident that carcinogenesis initiates from a particular subset of cells termed cancer stem cells (CSCs) which express particular surface markers and have stem-cell-like traits, including plasticity, quiescence, renewal, and drug resistance (Batlle and Clevers, 2017). Although the theory of CSCs has not yet been well-established, a plethora of studies have demonstrated that CSCs, or cancer cells with stem-cell-like properties, were more resistant to chemotherapy (Zhao, 2016). In EC, CSCs protect themselves against cytotoxic agents partly by facilitating the process of drug efflux. This self-protective mechanism is frequently found among side population (SP) cells, a subset of cells identified by flow cytometry that express specific surface markers (Hadnagy et al., 2006; Huang D. et al., 2009; Zhao et al., 2014). Although they are rare in TME, SP cells enrich in CSCs and express high levels of ATP-binding cassette (ABC) transporters, such as ABCG2 and ABCG5, which are responsible for drug efflux and multidrug resistance (Gottesman et al., 2002; Li et al., 2011). Studies demonstrated that expression of ABCG2 was significantly upregulated in esophageal CSCs, leading to the resistance to cisplatin and 5-fluorouracil, as well as offering a potential therapeutic target to avert chemoresistance (Cheng et al., 2012; Yue et al., 2015). Beyond this, esophageal CSCs may also have the ability to hinder the process of drug influx. A study demonstrated that p75 neurotrophin receptor (p75NTR) + cells have stem-cell-like properties, including cisplatin resistance, and this was possibly due to the downregulation of copper uptake protein 1 (CTR1), a major copper influx transporter in mammalian cells (Huang S. D. et al., 2009). Taken together, esophageal CSCs take advantage of the specific distribution of membrane transporters to maintain the intracellular drug concentration at a harmless level and avoid the cytotoxic effect of chemotherapy (Figure 1A).

The resistance mechanism of esophageal CSCs is driven by multiplecrucial signaling pathways (Figure 1A). Notch signaling is a cell-fate-determination pathway participating in various aspects of cell biology and interactions between cancer cells and TME (Meurette and Mehlen, 2018). Notch signaling also plays a vital role in the initiation and progression of EC (Song et al., 2014; Kunze et al., 2020). Notably, Notch signaling was demonstrated to enhance chemoresistance in both ESCC and EAC (Wang et al., 2014; Kunze et al., 2020). The aberrant activation of Notch signaling is partly owing to overexpression of Protein arginine methyltransferase 1 (PRMT1) which mediates mono and asymmetric dimethylation of the guanidino nitrogens of arginyl residues (Tang et al., 2000). Histone H4R3me2a mediated by PRMT1 promotes proliferation of CSCs and activates Notch and Wnt/β-catenin signaling, leading to enhanced drug resistance (Zhao et al., 2019a). Moreover, somatic mutations on the Notch1 gene were commonly found in patients with partial responses or stable diseases after neoadjuvant chemotherapy (Liu et al., 2020). Yes-associated protein (YAP), a Hippo pathway coactivator, confers stem-cell-like properties on EC cells by upregulating SOX9 (Wang L. et al., 2019). Notably, YAP induces the expression of EGFR, which is associated with the resistance to 5-fluorouracil and docetaxel (Song et al., 2015). Inhibition of YAP can reduce stem-cell-like properties and is a potential therapeutic strategy to attenuate drug resistance in EC. In addition, the maintenance of drug resistance in esophageal CSCs also depends on the activation of various critical signaling pathways, such as Wnt/catenin, TGF, and hedgehog pathway (Liu et al., 2017; Wang D. et al., 2019; Zhao et al., 2019b).

Like other cancers, the identification of esophageal CSCs is based on the specific expression of stemness-related surface markers, including CD24, CD44, CD90, CD133, CLDN4, EpCAM, OV6, and p75NTR, some of which are associated with drug resistance and have the potential to predict the therapeutic response (Yamaguchi et al., 2016; Jiménez et al., 2017; Liu et al., 2017; Wang et al., 2017; Wang D. et al., 2019; Sun et al., 2018; Xu et al., 2018; Lin et al., 2019; Figure 1A). p75NTR, also referred to as CD271, is a receptor of the neurotrophic growth factor family that mediates various cell outcomes, such as cell apoptosis during neurodevelopmental processes (Barker, 2004). In EC, p75NTR is a specific marker for CSCs at mitotic quiescent periods; p75NTR + cells exhibit enhanced drug resistance and have the potential to serve as therapeutic targets (Huang S. D. et al., 2009; Yamaguchi et al., 2016). OV6 + is a potential marker for esophageal CSCs, which has been demonstrated to be associated with drug resistance (Wang et al., 2017). In OV6 + cells, autophagy is significantly activated to maintain stem-cell-like properties, including drug resistance, by stabilizing ATG7-dependent B-catenin. Beyond these, CLDN4 is a CSC marker that has the potential to predict therapeutic response after chemotherapy, which is of great clinical importance to select proper candidates for chemotherapy (Lin et al., 2019). Nevertheless, most of the studies to date only focused on demonstrating the guilt-by-association between drug resistance and specific expression of surface markers. In-depth analyses regarding the causal association and clinical applicability of CSC markers in the chemotherapeutic setting are urgently needed.

### Cancer-Associated Fibroblasts

Cancer-associated fibroblasts (CAFs), characterized by high expression of α-smooth muscle actin and fibroblast activation protein-α, represent a dominant component of tumor stroma in the TME and play prominent functional roles in cancer progression and drug resistance (Kalluri, 2016). Normal fibroblasts (NFs) are usually quiescent and can be activated in response to specific circumstances, such as wound healing, leading to increased production of TGF-β and a highly contractile phenotype (Rockey et al., 2013). Like many other cancers, the tumorigenesis of EC is associated with chronic inflammatory and mucosal injury. Mediated by functional molecules, such as microRNAs and lncRNAs, NFs are transformed into CAFs and confer drug resistance on surrounding EC cells by secreting soluble factors and stimulating pro-tumorigenic signals (Tanaka et al., 2015; Tong et al., 2020). Interleukin 6 (IL-6), a multifunctional cytokine, not only mediate immune and inflammatory response but also participate in various hallmarks of cancer, including drug resistance. CAFs are major sources of IL-6 in the TME, which enhance the chemoresistance of ESCC cells by upregulating C-X-C motif chemokine receptor 7 (CXCR7) through STAT3/NF-κB pathway (Qiao et al., 2018). CAFs-derived IL-6 also confers resistance to chemoradiotherapy on EAC patients. Interestingly, although serum IL-6 cannot stratify patients with different response to neoadjuvant chemoradiotherapy, circulating ADAM12 is significantly associated with poor response to chemoradiation, indicating its potential to predict therapeutic response in patients with EAC (Ebbing et al., 2019). Plasminogen activator inhibitor-1 (PAI-1) is a well-known cytokine that functions as a principal inhibitor of vascular fibrinolysis (Ghosh and Vaughan, 2012). Cisplatin-induced DNA damage in CAFs promotes the paracrine of PAI-1 and activate AKT and ERK1/2 pathway in EC cells, eventually leading to enhanced cancer cell proliferation and reduced cytotoxic effect of cisplatin (Che et al., 2018). Furthermore, TGF-β signaling is involved in crosstalk between cancer cells and CAFs that protect ESCC cells from several conventional chemotherapeutic agents, likely due to the transcriptional activation induced by FOXO1 which can stimulate TGF-β1 promoter activity (Zhang et al., 2017). In the context of molecular targeted therapy, FGF in fibroblast supernatant may play a role in attenuating the effect of lapatinib on ESCC cells, which can be abrogated by additionally treating it with FGFR inhibitor (Saito et al., 2015). Taken together, the CAF secretome, as well as corresponding receptors on cancer cells, represents attractive therapeutic targets that hold the promise to address drug resistance in a combined manner (Figure 1B).

### Immune Inflammatory Cells

The programmed death 1 (PD-1) pathway serves as a critical immune checkpoint to limit immune responses mediated by T cells in the TME. Tumor cells can evade the immune responses by two ligands, programmed death ligand 1 (PD-L1) and programmed death ligand 2 (PD-L2), both of which engage the PD-1 receptor and inhibit T-cell activation, known as tumor immune evasion (Freeman et al., 2000; Latchman et al., 2001; Juneja et al., 2017; Dong et al., 2018; Zhao and Huang, 2020). The discovery of tumor immune evasion paves the way to treat cancer in an immune-checkpoint-based manner, which is now one of the most promising therapeutic strategies for various types of cancer (Hamid et al., 2013; Borghaei et al., 2015; Nanda et al., 2016). Currently, immune checkpoint inhibitors, represented by nivolumab and pembrolizumab, have achieved initial success in the treatment of advanced or refractory EC (Hong and Ding, 2019; Kato et al., 2019; Shah et al., 2019). Due to low response rates among EC patients, however, further efforts are needed to elucidate the resistance mechanism of immunotherapy. Among all the patients with EC, less than 20% of them express PD-L1 (Okadome et al., 2020), which means a certain number of patients with EC are hard to get any therapeutic benefit from immune checkpoint inhibitors. Moreover, the expression of PD-L1 on the cell surface was found to be highly heterogeneous in EC (Yan et al., 2019). These facts are responsible for the low response rates of immunotherapy. On the other hand, although immune inflammatory cells infiltrating in the TME are considered as a double-edged sword for tumor progression (Hanahan and Weinberg, 2011), the lower status of tumor-infiltrating lymphocytes (TILs) has been demonstrated to be associated with unfavorable clinical outcomes of patients with EC (Yagi et al., 2019a; Däster et al., 2020). To overcome these challenges, identifying reliable biomarkers to select proper populations who can get the maximum therapeutic benefit is of great clinical importance. Notably, the combination of PD-L1 expression and TILs status has the potential to serve as predictive biomarkers for patients with EC (Yagi et al., 2019a). PD-L2 has also been demonstrated to be associated with a worse prognosis in EC (Ohigashi et al., 2005; Okadome et al., 2020). The identification of these biomarkers contributes not only to prognosis prediction but also to patient classification and selection (Baba et al., 2020). Based on PD-L1 expression and T-cell infiltration, a cancer stratification model including four types of TME status has been proposed to tailor ideal immune-based therapeutic strategy (Teng et al., 2015).

Furthermore, combining immunotherapy with conventional therapy has the potential to overcome drug resistance and provide better therapeutic benefits (Gotwals et al., 2017; Kelly et al., 2018). EC cell lines treated with 5-fluorouracil exhibits a high level of PD-L1, which provides factual bases for the combination of chemotherapy and immunotherapy to some extent (Van Der Kraak et al., 2016). It is also evident that neoadjuvant chemotherapy could induce CD4 and CD8 T cells in the TME of EC (Tsuchikawa et al., 2012). Moreover, a recent study found that paclitaxel-nedaplatin could induce the reconstruction of TILs, which was partly due to the migration of T cells from peripheral blood to the TME (Zhang et al., 2018). These findings further unraveled the modulatory role of chemotherapy in T cell immune response and provided theoretical bases for the rational combination of chemotherapy and immunotherapy. That is, in addition to their cytotoxicity, some chemotherapeutic agents also have the potential to be used as sensitizers for immunotherapy. Importantly, adverse events (AEs) must be taken into account, especially in the context of combined therapy. Beyond these, inducing anti-tumor immune responses by cancer vaccines is another therapeutic strategy. A recent clinical trial reported that vaccination with the CPV S-588410 induced functional CD8 + and CD4 + TILs, as well as PD-L1 expression, in EC, indicating that S-588410 vaccine combined with PD-L1 inhibitors might be an effective therapeutic option (Daiko et al., 2020; Figure 1C). Moreover, a recent study found that high density of tumor-associated macrophages (TAMs) was associated with increased PD-L1 expression and a worse outcome in EC, indicating a rational combination therapy targeting TAMs and PD-L1 (Yagi et al., 2019b).

## Non-Cellular Components

### Hypoxia

**FIGURE 2:**
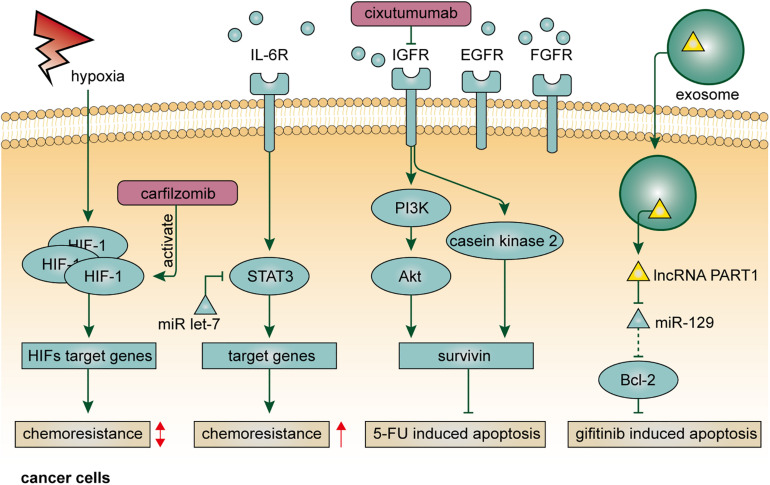
Resistance mechanisms of non-cellular components in the tumor microenvironment in EC.

Within the TME, the formation of the hypoxic region is usually associated with the imbalance between the rapid expansion of solid tumors and abnormal structure and function of tumor vasculature (Jain, 2005). Insufficient blood supply, on the one hand, influences the effective delivery of antitumor drugs, and on the other hand, alters the local concentration of oxygen and other nutrients, resulting in compromised metabolism and reduced drug sensitivity in cancer cells (Tang et al., 2020). Hypoxia confers drug resistance through various signaling pathways involved in apoptosis, autophagy, DNA damage repair, mitochondrial activity, p53, and drug efflux (Graeber et al., 1996; Jing et al., 2019). Hypoxia-inducible factors (HIFs) are transcription factors that represent the pivotal mediator of the hypoxic response in the cellular microenvironment and play key roles in resistance to conventional anticancer therapy (Rohwer and Cramer, 2011). In EC, expression of HIF-1 is correlated with venous invasion, VEGF expression, and microvessel density (Kimura et al., 2004). Alleviation of the hypoxic condition in EC is usually accompanied by downregulation of HIF-1 expression and complete response to chemotherapy (Lee et al., 2015). Clinically, combined with p53 and p21, overexpression of HIF-1 is a sensitive indicator to predict treatment response after chemoradiotherapy (Sohda et al., 2004). However, the effect of HIF-1 on drug resistance in EC is likely bidirectional (Figure 2). By single-cell RNA-seq, a recent study found that paclitaxel-resistant EC cells were characterized with lower expression of HIF-1 signaling genes, and that the chemoresistance could be attenuated through activating HIF-1 signaling by using carfilzomib, indicating a rational therapeutic combination of carfilzomib and paclitaxel (Wu et al., 2018).

### Cytokines

Cytokines serve important roles in intra- or intercellular signal transduction by autocrine, paracrine, and endocrine fashions. IL-6 is a principal mediator involved in the acute-phase response to injury and infection (Xing et al., 1998). Besides its pro-inflammatory functions, IL-6 also plays a crucial role in drug resistance in human cancer. As mentioned above, IL-6 derives from stromal cells in the TME, such as CAFs, and confer chemoresistance on EC cells via multiple pathways (Qiao et al., 2018; Ebbing et al., 2019). Indeed, the upregulation of IL-6 can be found in Barrett’s esophagus, a widely accepted precancerous lesion of EAC, and enables esophageal epithelia resistant to cell apoptosis, leading to a higher risk of carcinogenesis (Dvorakova et al., 2004). EC cells treated with cisplatin exhibit higher expression of IL-6, which promotes phosphorylation of STAT3 and thereby confers cancer hallmarks, including evasion of apoptosis and chemoresistance, on themselves or surrounding EC cells in autocrine or paracrine manners (Sugimura et al., 2012). Notably, microRNA let-7 can restore the efficacy of chemotherapy by targeting IL-6/STAT3 prosurvival pathway activated by cisplatin (Figure 2). Therefore, treating with let-7 may have the potential to prolong the duration of cytotoxic effect in the TME, and the functional role of let-7 in tumor-associated stromal cells requires further investigations. In addition to IL-6, the serum level of IL-6R is also associated with chemoresistance (Makuuchi et al., 2013). EC patients with an elevated level of serum IL-6R are more resistant to neoadjuvant chemoradiotherapy, indicating the clinical value of IL-6R to serve as a biomarker for patient selection. IL-1β, another member of the interleukin family, promotes tumor development by driving chronic inflammation, tumor angiogenesis, and induction of immunosuppressive cells (Bent et al., 2018). In EC, IL-1β expression was found to be correlated with chemoradiotherapy response (Chen et al., 2012). The same study also demonstrated that caffeic acid phenethyl ester (CAPE), a pro-inflammatory natural chemical compound that can specifically block NF–κB and attenuate IL-1β expression, increased the sensitivity of EC cells to cisplatin. However, to date, the resistance mechanism of IL-1β in EC still lacks in-depth investigation.

### Growth Factors

Growth factors typically function as signaling molecules that mediate intercellular communication and trigger various critical cellular processes, such as cell proliferation and differentiation (Werner and Grose, 2003; Hicklin and Ellis, 2005; Normanno et al., 2006; Pollak, 2008; Turner and Grose, 2010). Unfortunately, in the context of TME, the blocking effect of growth factors on cell apoptotic pathways leads to stronger resistance to anticancer drugs, since the cell apoptotic program is considered a natural barrier preventing normal cells transform into malignancy (Adams and Cory, 2007). In EC, IGF-1 inhibits cell apoptosis induced by a variety of common chemical agents, including cisplatin, 5-fluorouracil, and camptothecin (Liu et al., 2002). Mechanistically, IGF-1, partly induced by Id1, can upregulate the expression of survivin via PI3K/Akt and casein kinase 2 signaling pathways, leading to inhibition of Smac/DIABLO release and activation of caspases, which are responsible for 5-fluorouracil-induced cell apoptosis (Juan et al., 2011; Li et al., 2014, 2016; Figure 2). The secretion of IGF-1 depends on both autocrine and paracrine manners. Blocking IGF-1R may be a useful method to not only retard tumor growth, but also make EC cells more sensitive to chemotherapy. Indeed, cixutumumab, a monoclonal antibody against IGF-1R, was demonstrated to significantly inhibited EC progression and metastasis, as well as chemoresistance to cisplatin and 5-fluorouracil (Li et al., 2014). Targeting IGF-2 or PI3K/Akt pathway is also a promising way to enhance chemosensitivity in EC. A study demonstrated that IGF-2-neutralizing antibody and PI3K/Akt pathway inhibitors could inhibit capacities of self-renew and chemoresistance to 5-fluorouracil in CD133-positive esophageal CSCs (Xu et al., 2018). Apart from the IGF family, EGFR and FGFR also contribute to drug resistance in EC. As mentioned above, YAP1 can transcriptionally upregulate EGFR, which is of importance to chemoresistance in EC (Song et al., 2015). Targeting YAP1 by verteporfin reduces the expression of YAP and EGFR and makes EC cells more sensitive to cytotoxic agents. Moreover, FGFR inhibitors can reduce FGF-mediated lapatinib resistance, although the mechanism is as yet unknown (Saito et al., 2015).

### Exosomes

Exosomes are a category of extracellular vesicles comprising various bioactive molecules, such as proteins, lipids, and nucleic acids. Recent advances in exosome-based biology processes have opened up a whole new range of intercellular communications within the TME (Valadi et al., 2007). Typically, functional molecules derived from host cells can invade nearby recipient cells through exosome-based transfer, resulting in the diffusion of malignant hallmarks (Melo et al., 2014). In EC, microRNAs and lncRNAs usually take advantage of this mode of action to facilitate drug resistance. For example, miRNA-193, one of the upregulated microRNAs in exosomes, was demonstrated to attenuate cisplatin-induced cell cycle inhibition to enhance chemoresistance in EC (Shi et al., 2020). Moreover, as mentioned above, EC-cell-derived lncRNA POU3F3 could enter into NFs via exosome-based transfer to induce their activation, which enhanced cisplatin resistance in EC in an IL-6-dependent manner (Tong et al., 2020). In terms of molecular targeted therapy, exosome-mediated transfer of lncRNA PART1 could competitively bind to miRNA-129 in recipient EC cells to upregulate Bcl-2 and inhibit cell apoptosis mediated by Bax, caspase-3, and c-PARP, leading to gefitinib resistance (Kang et al., 2018; Figure 2). Due to intratumoral heterogeneity, the sensitivity of cancer cells to therapeutic regimens is diverse. The spread of drug resistance in the TME is, at least in part, ascribed to exosome-mediated intercellular communication.

## Conclusion and Future Direction

It is paramount to understand the underlying mechanism of drug resistance in EC, of which the TME is an indispensable participant. TME is a dynamic and ever-changing complex biological network, whose diverse cellular and non-cellular components influence specific traits and fates of tumor cells from the outside, and this is responsible for the development of resistance to conventional therapeutic agents to some extent. It is now evident that the formation of drug resistance in EC is a multifactorial complex process involving not only cancer cells but CSCs, tumor-associated stromal cells, hypoxia, soluble factors, extracellular vesicles, etc. CSCs, with multiple upregulated stemness markers and activated pro-survival signaling pathways, are a major component of resistant subpopulations in the TME. Given that chemotherapy or molecular targeted therapy are difficult to fully eradicate CSCs, poor pathological response and tumor relapse can be considered as inevitable posttreatment events. Autocrine and paracrine activities of CAFs and exosome-mediated intercellular communication can exacerbate the spread of drug resistance from resistant cells to sensitive cells. This multipath-dependent resistance makes treatment even trickier.

Looking forward, although adverse events are inevitable, combination therapy still represents a promising strategy to overcome the obstacle. By targeting crucial molecules in the TME, different combinations among chemotherapy, molecular targeted therapy, and immunotherapy hold the promise to thoroughly eradicate EC cells, which require a more comprehensive elucidation of the TME in EC. The heterogeneity within the TME, as well as individual differences, limit the general implementation of antitumor drugs to clinical scenarios. Therefore, it is critical to identify reliable microenvironmental biomarkers that can predict therapeutic response before initial treatment. Although conventional therapy, including surgery, radiotherapy, and chemotherapy are still predominant in the clinical management of EC, an increasing number of preclinical and clinical research will hopefully translate to novel, safe, and effective clinical treatment options in the foreseeable future.

## Author Contributions

YZ and YY conceptualized the study, revised the manuscript, and supervised the study. SL and XZ conceptualized the study, drafted the manuscript, and made the figures. CZ, JQ, YSY, CM, XX, and JZ collected the literature and revised the manuscript. All authors read and approved the final manuscript.

## Conflict of Interest

The authors declare that the research was conducted in the absence of any commercial or financial relationships that could be construed as a potential conflict of interest.
