# Supplementary figures and images for: Advances in Drug Resistance of Esophageal Cancer: From the Perspective of Tumor Microenvironment
Source: Front Cell Dev Biol. 2021 Mar 19;9:664816. doi: 10.3389/fcell.2021.664816 (PMC8017339; doi:10.3389/fcell.2021.664816)

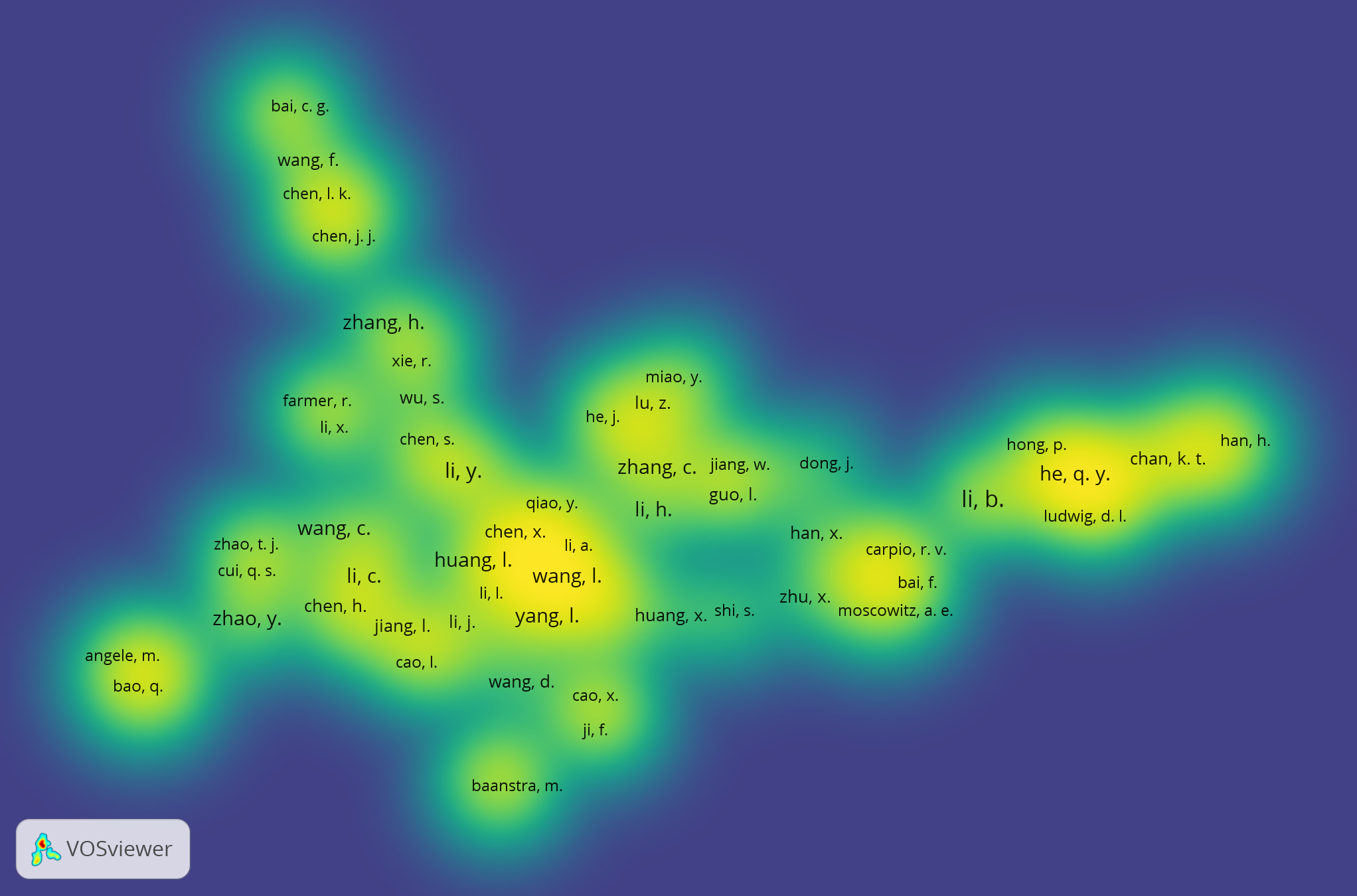

Supplement: Supplementary file 1 [file Image_1.PNG]

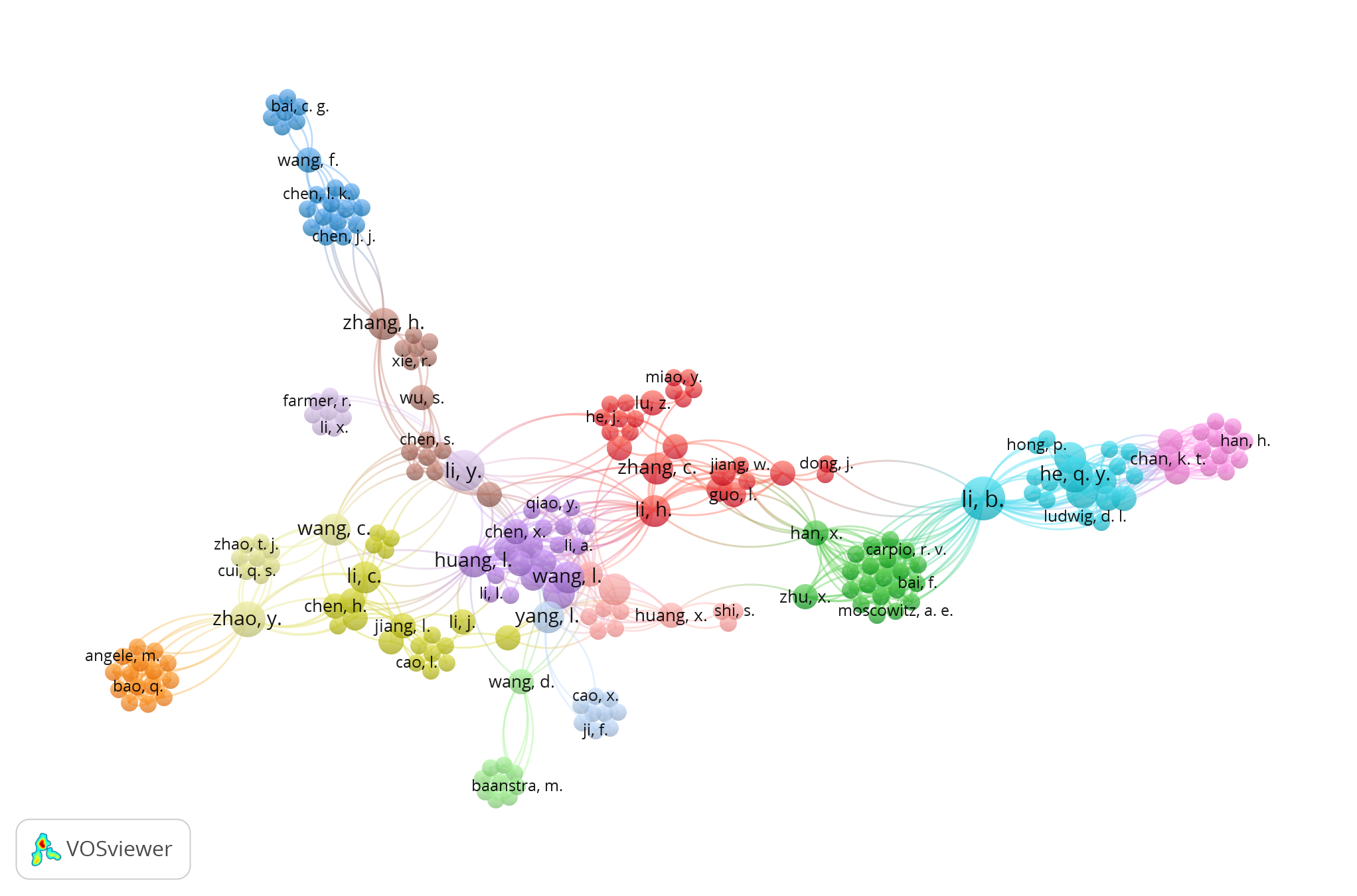

Supplement: Supplementary file 2 [file Image_2.PNG]

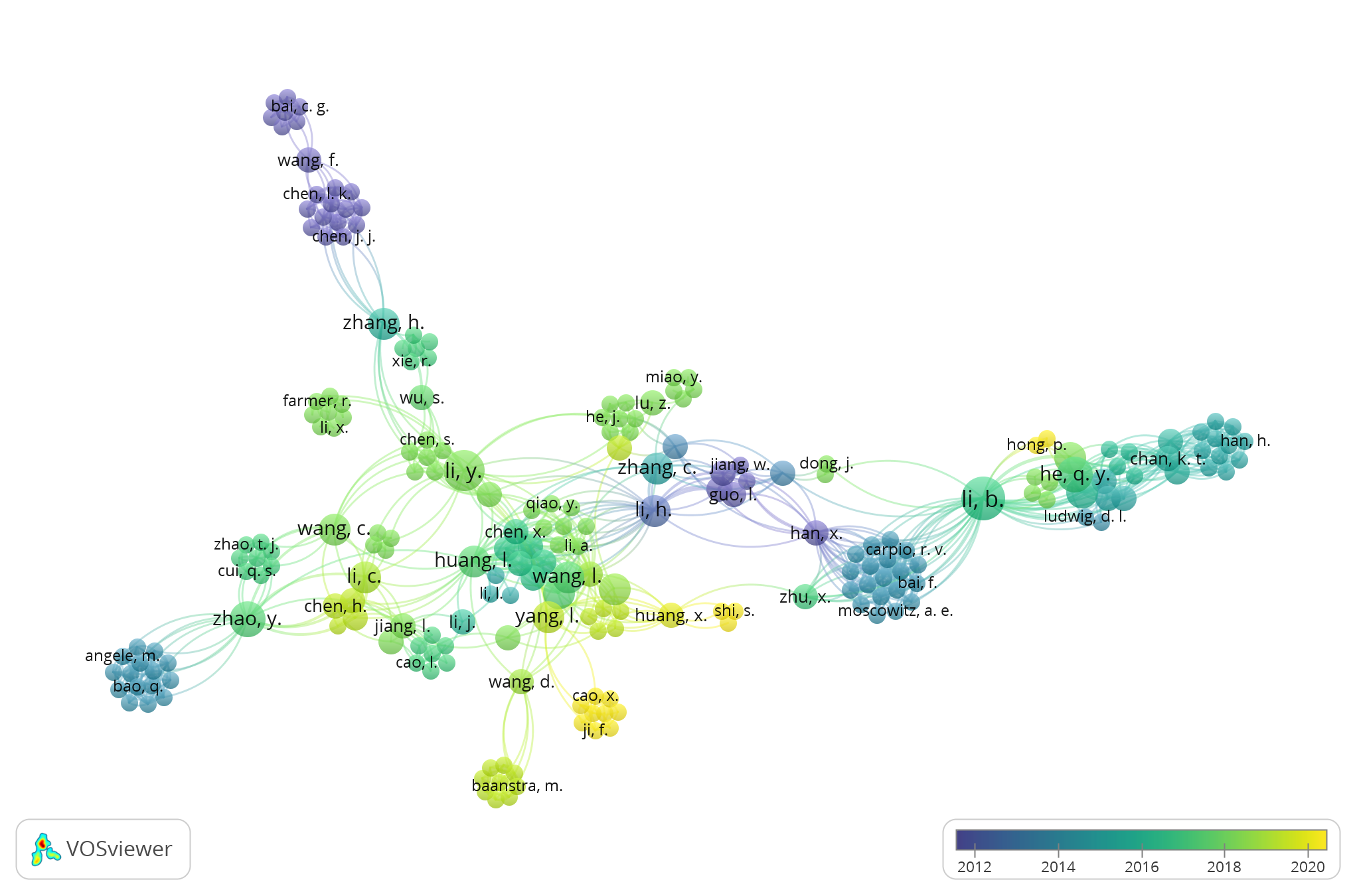

Supplement: Supplementary file 3 [file Image_3.PNG]
